# Supplementary material for: Increased Trypanocidal Activity of the Salinomycin Derivative Ironomycin Is Due to ROS Production and Iron Uptake Impairment
Source: Molecules. 2024 Nov 27;29(23):5597. doi: 10.3390/molecules29235597 (PMC11643196; doi:10.3390/molecules29235597)
Supplement: Supplementary file 1 [file molecules-29-05597-s001.zip › molecules-3118645-supplementary.pdf]

molecules

Supplementary Materials

Increased trypanocidal activity of the salinomycin derivative ironomycin is due to ROS production and iron uptake impairment

Dietmar Steverding [1,*], Stuart A. Rushworth [1], Georgina R. Hurle [2], Michał Antoszczak [3], Michał Sulik [3] Adam Huczyński [3] and Kevin M. Tyler [2]

[1] Bob Champion Research and Education Building, Norwich Medical School, University of East Anglia, Norwich NR4 7UQ, United Kingdom; D.Steverding@uea.ac.uk (D.S.); S.Rushworth@uea.ac.uk (S.A.R.)
[2] BioMedical Research Centre, Norwich Medical School, University of East Anglia, Norwich NR4 7TJ, United Kingdom; G.Hurle@uea.ac.uk (G.R.H.); K.Tyler@uea.ac.uk (K.M.T.)
[3] Department of Medical Chemistry, Faculty of Chemistry, Adam Mickiewicz University, Uniwersytetu Poznańskiego 8, 61–614 Poznań, Poland; michal.antoszczak@amu.edu.pl (M.A.); michal.sulik@amu.edu.pl (M.S.); adam.huczynski@amu.edu.pl (A.H.)
* Correspondence: D.Steverding@uea.ac.uk

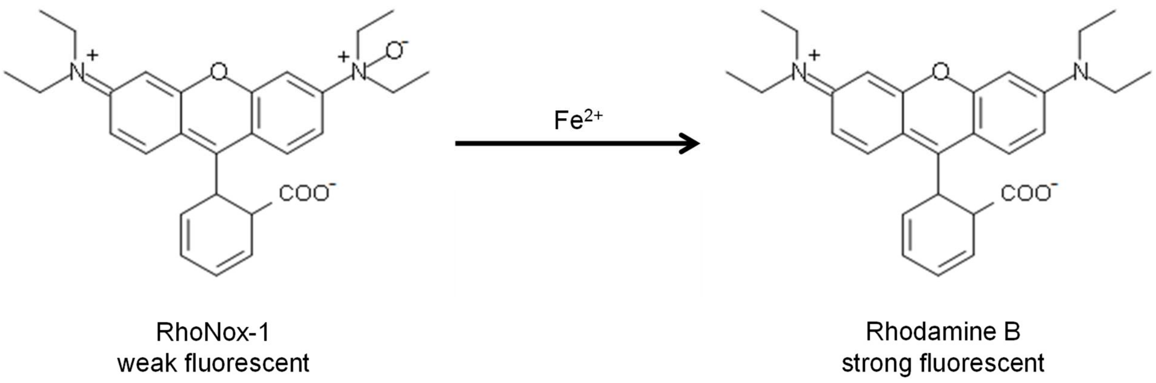

Scheme S1. Reduction of RhoNox-1 by iron(II) to rhodamine B.

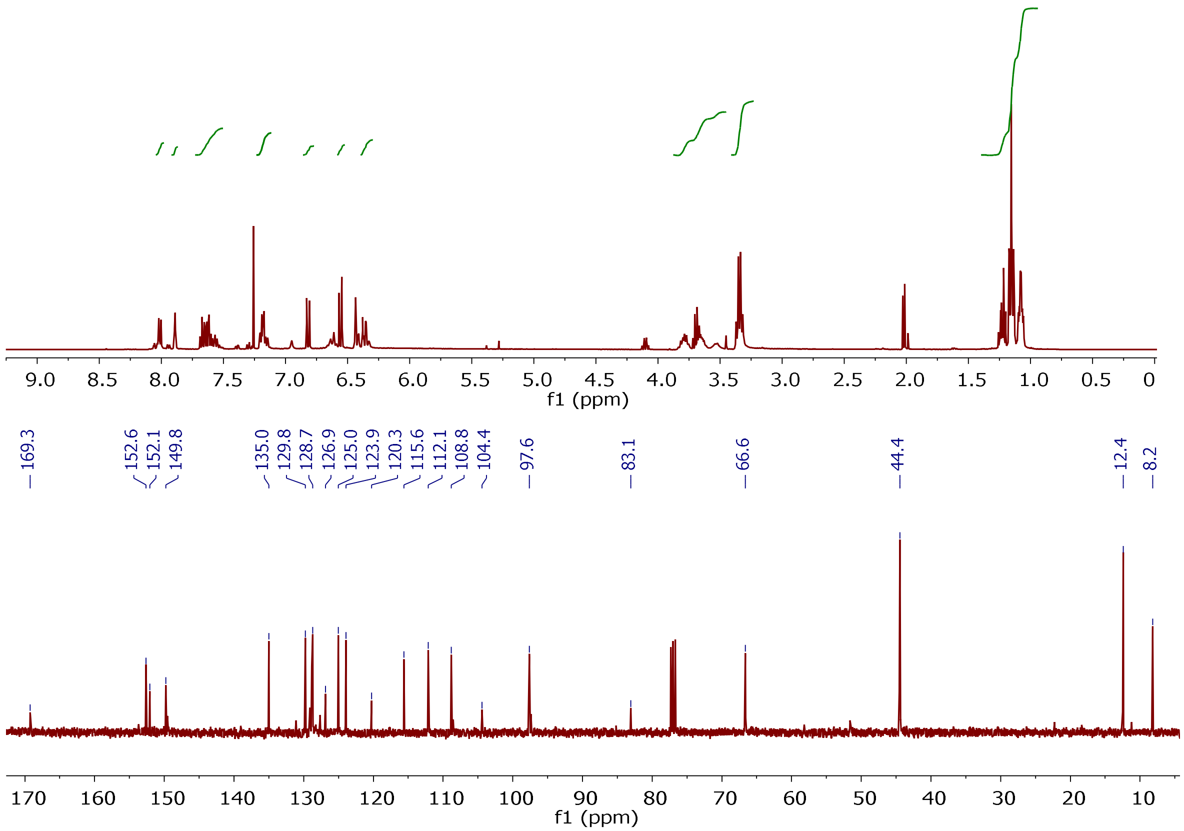

Figure S1. ^{1}H-NMR (top) and ^{13}C-NMR (bottom) spectra of RhoNox-1 in $CDCl_3$..
